# First-line high-dose sequential chemotherapy with rG-CSF and repeated blood stem cell transplantation in untreated inflammatory breast cancer: toxicity and response (PEGASE 02 trial)

**DOI:** 10.1038/sj.bjc.6690714

**Published:** 1999-10

**Authors:** P Viens, T Palangié, M Janvier, M Fabbro, H Roché, T Delozier, J P Labat, C Linassier, B Audhuy, F Feuilhade, B Costa, R Delva, H Cure, F Rousseau, A Guillot, M Mousseau, J M Ferrero, V J Bardou, J Jacquemier, P Pouillart

**Affiliations:** Institut Paoli Calmettes, 232 Blvd Sainte Marguerite, Marseille, Cedex 09, 13273, France; Institut Curie, Paris, France; Centre René Huguenin, Saint-Cloud, France; Centre Val d'Aurelle, Montpellier, France; Centre Claudius Regaud, Toulouse C, France; Centre François Baclesse, Caen, France; CHU A Morvan, Brest, France; Hôpital Bretonneau, Tours, France; Hôpital Pasteur, Colmar, France; CHU Henri Mondor, Créteil, France; Institut Jean Godinot, Reims, France; Centre Paul Papin, Angers, France; n, Centre Jean Perri, Clermont-Ferrand, France; Centre René Dubos, Pontoise, France; Hôpital Nord, Saint-Etienne, France; CHU A Michallon, Grenoble, France; Centre Antoine Lacassagn, Nice, France

**Keywords:** inflammatory breast cancer, high-dose sequential chemotherapy

## Abstract

Despite the generalization of induction chemotherapy and a better outcome for chemosensitive diseases, the prognosis of inflammatory breast cancer (IBC) is still poor. In this work, we evaluate response and toxicity of high-dose sequential chemotherapy with repeated blood stem cell (BSC) transplantation administered as initial treatment in 100 women with non-metastatic IBC. Ninety-five patients (five patients were evaluated as non-eligible) of median age 46 years (range 26–56) received four cycles of chemotherapy associating: cyclophosphamide (C) 6 g m^−2^ – doxorubicin (D) 75 mg m^−2^ cycle 1, C: 3 g m^−2^ – D: 75 mg m^−2^ cycle 2, C: 3 g m^−2^ – D: 75 mg m^−2^ – 5 FU 2500 mg m^−2^ cycle 3 and 4. BSC were collected after cycle 1 or 2 and reinfused after cycle 3 and 4. rG-CSF was administered after the four cycles. Mastectomy and radiotherapy were planned after chemotherapy completion. Pathological response was considered as the first end point of this trial. A total of 366 cycles of chemotherapy were administered. Eighty-seven patients completed the four cycles and relative dose intensity was respectively 0.97 (range 0.4–1.04) and 0.96 (range 0.25–1.05) for C and D. Main toxicity was haematological with febrile neutropenia ranging from 26% to 51% of cycles; one death occurred during aplasia. Clinical response rate was 90% ± 6%. Eighty-six patients underwent mastectomy in a median of 3.5 months (range 3–9) after the first cycle of chemotherapy; pathological complete response rate in breast was 32% ± 10%. All patients were eligible to receive additional radiotherapy. High-dose chemotherapy with repeated BSC transplantation is feasible with acceptable toxicity in IBC. Pathological response rate is encouraging but has to be confirmed by final outcome. © 1999 Cancer Research Campaign


*British Journal of Cancer* (1999) **81**(5), 449–456
© 1999 Cancer Research Campaign
Article no. bjoc.1999.0714

### First-line high-dose sequential chemotherapy with rG-CSF and repeated blood stem cell transplantation in untreated inflammatory breast cancer: toxicity and response (PEGASE 02 trial)

**P Viens[1], T Palangié[2], M Janvier[3], M Fabbro[4], H Roché[5], T Delozier[6], JP Labat[7], C Linassier[8], B Audhuy[9], F Feuilhade[10], B Costa[11], R Delva[12], H Cure[13], F Rousseau[14], A Guillot[15], M Mousseau[16], JM Ferrero[17], VJ Bardou[1], J Jacquemier[1] and P Pouillart[2]**

[1]Institut Paoli Calmettes, 232 Blvd Sainte Marguerite, 13273 Marseille, Cedex 09 France; [2]Institut Curie, Paris, France; [3]Centre René Huguenin, Saint-Cloud, France; [4]Centre Val d'Aurelle, Montpellier, France; [5]Centre Claudius Regaud, Toulouse, C France; [6]Centre François Baclesse, Caen, France; [7]CHU A Morvan, Brest, France; [8]Hôpital Bretonneau, Tours, France; [9]Hôpital Pasteur, Colmar, France; [10]CHU Henri Mondor, Créteil, France; [11]Institut Jean Godinot, Reims, France; [12]Centre Paul Papin, Angers, France; [13]Centre Jean Perrin, Clermont-Ferrand, France; [14]Centre René Dubos, Pontoise, France; [15]Hôpital Nord, Saint-Etienne, France; [16]CHU A Michallon, Grenoble, France; [17]Centre Antoine Lacassagne, Nice, France

**Summary** Despite the generalization of induction chemotherapy and a better outcome for chemosensitive diseases, the prognosis of inflammatory breast cancer (IBC) is still poor. In this work, we evaluate response and toxicity of high-dose sequential chemotherapy with repeated blood stem cell (BSC) transplantation administered as initial treatment in 100 women with non-metastatic IBC. Ninety-five patients (five patients were evaluated as non-eligible) of median age 46 years (range 26–56) received four cycles of chemotherapy associating: cyclophosphamide (C) 6 g $m^{-2}$ – doxorubicin (D) 75 mg $m^{-2}$ cycle 1, C: 3 g $m^{-2}$ – D: 75 mg $m^{-2}$ cycle 2, C: 3 g $m^{-2}$ – D: 75 mg $m^{-2}$ – 5 FU 2500 mg $m^{-2}$ cycle 3 and 4. BSC were collected after cycle 1 or 2 and reinfused after cycle 3 and 4. rG-CSF was administered after the four cycles. Mastectomy and radiotherapy were planned after chemotherapy completion. Pathological response was considered as the first end point of this trial. A total of 366 cycles of chemotherapy were administered. Eighty-seven patients completed the four cycles and relative dose intensity was respectively 0.97 (range 0.4–1.04) and 0.96 (range 0.25–1.05) for C and D. Main toxicity was haematological with febrile neutropenia ranging from 26% to 51% of cycles; one death occurred during aplasia. Clinical response rate was 90% ± 6%. Eighty-six patients underwent mastectomy in a median of 3.5 months (range 3–9) after the first cycle of chemotherapy; pathological complete response rate in breast was 32% ± 10%. All patients were eligible to receive additional radiotherapy. High-dose chemotherapy with repeated BSC transplantation is feasible with acceptable toxicity in IBC. Pathological response rate is encouraging but has to be confirmed by final outcome.
© 1999 Cancer Research Campaign

**Keywords:** inflammatory breast cancer; high-dose sequential chemotherapy

Inflammatory breast cancer (IBC) is an uncommon disease, occurring in about 2–4% of all breast cancer (Jaiyesimi et al, 1992). It is generally defined as a clinical entity, corresponding to the T4d stage of the 1988 UICC classification.

Despite its low frequency, IBC remains a challenge for oncologists. When treated with surgery or radiotherapy alone, or both, 5-year survival did not exceed 15% (Swain and Lippman, 1989). Generalization of neoadjuvant chemotherapy has largely improved treatment of IBC, but prognosis is still very poor, with 5-year survival rates between 30 and 50% (Rouëssé et al, 1986; Bauer et al, 1995), and there is no current consensus on the 'best' induction chemotherapy regimen. Response to initial chemotherapy has been described as predictive of outcome, with progression-free and overall survival significantly higher for responding patients than for non-responding patients (Chevallier et al, 1987; Palangie et al, 1994). Achievement of complete pathological response seems a particularly important prognostic factor (Feldman et al, 1986; Noguchi et al, 1988; Maloisel et al, 1990; Armstrong et al, 1993; Sataloff et al, 1995). We can therefore speculate that improving efficacy of first-line chemotherapy could be one method of improving IBC prognosis.

Based on in vitro and animal models (Frei and Canellos, 1980; Griswold et al, 1987), showing dose–response relationship for several anticancer drugs, especially alkylating agents, and retrospective studies evaluating the impact of dose intensity in breast cancer (Hryniuk and Bush, 1984), a number of pilot studies have tested the impact of dose escalation, including high-dose chemotherapy with stem cell transplantation in breast cancer (Antman and Gale, 1988; Peters et al, 1993). Large studies have been done in metastatic or non-metastatic poor prognosis breast cancer, in which high-dose chemotherapy was generally performed after induction by conventional chemotherapy, and in selected patients with responding disease in metastatic situation. This strategy allows delivery of very high-dose chemotherapy, generally alkylating agents, in a single course using the concept of dose effect.

*Received 24 February 1998*
*Revised 25 March 1999*
*Accepted 12 April 1999*

*Correspondence to:* P Viens

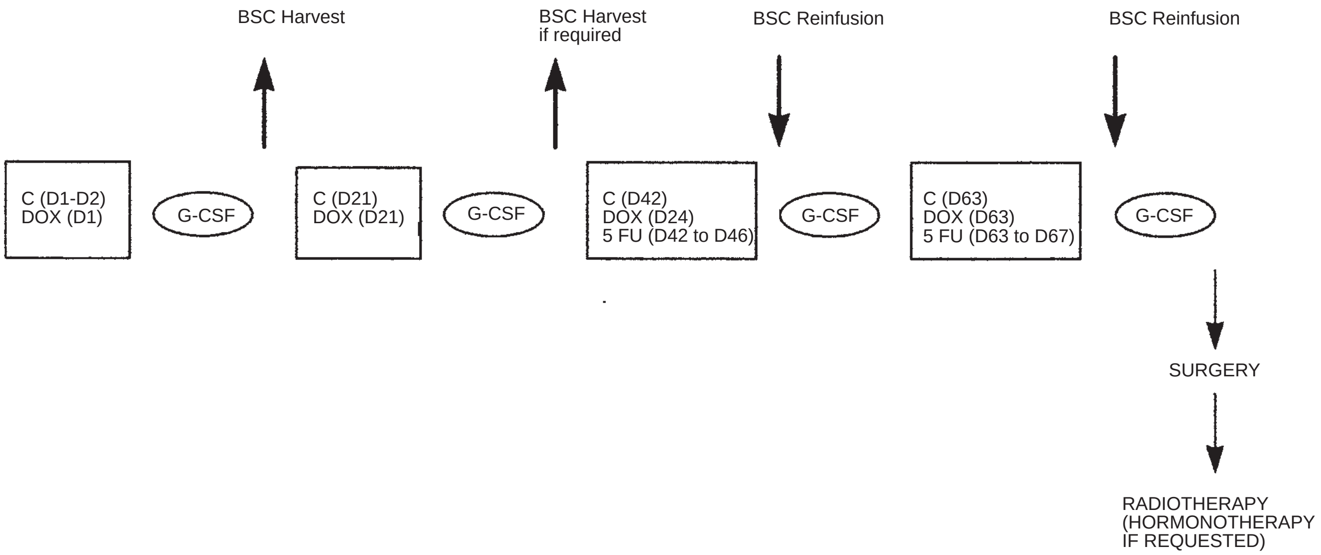

**Figure 1** Pegase 02 regimen. C = Cyclophosphamide: 3 g $m^{-2}$ $day^{-1}$. Dox = Doxorubicin: 75 mg $m^{-2}$. 5-FU = Fluorouracil: 500 mg $m^{-2}$ $day^{-1}$ continuous infusion. BSC: Blood stem cells. D: day.

Another possibility of intensifing chemotherapy is to increase doses along multiple cycle, using the concept of dose intensity in first-line treatment. The use of haematopoietic growth factors and/or peripheral stem cells allows regimen to be designed (Basser et al, 1995; Swain et al, 1996; Shipp et al, 1995; Stoppa et al, 1997; Viens et al, 1997) in which dose and dose intensity are significantly increased. These can then be given safely, as out patient first-line treatment with the objective of improving chemotherapy response and disease prognosis in the whole patient population.

Based on those considerations, the High Dose Chemotherapy for Breast Cancer Study Group (PEGASE) of the French Federation of Anticancer Centers initiated such a study (PEGASE 02) aimed at evaluating: toxicity and feasibility of high-dose sequential chemotherapy with rG-CSF (filgrastim) and stem cell support in inflammatory breast cancer, response to this chemotherapy with emphasis on pathological response and, secondary, impact on disease-free survival and survival. We report here toxicity and response rate.

### MATERIALS AND METHODS

### Eligibility

One hundred consecutive women with primary inflammatory breast cancer were included in this study. Inflammatory breast cancer was defined as follows: histologically documented adenocarcinoma of the breast with inflammatory signs (erythema, 'peau d'orange' appearance and increase in local temperature) which involved ≥ one-third of the breast (T4d of the 1988 International Union Against Cancer [UICC] classification). Absence of dermal lymphatic carcinomatosis was not a criterion for exclusion.

Other inclusion criteria were: age between 18 and 60 years, WHO performance status ≤ 2, no previous history of malignancy, normal cardiac function assessed by a normal ECG and normal left ventricular ejection fraction estimated by echocardiography or radionuclide cardiac scan, polynuclear neutrophil count greater than $1.5 \times 10^9$ $l^{-1}$, platelet count superior to $100 \times 10^9$ $l^{-1}$, total bilirubin, serum creatinin, ASAT and ALAT inferior to 1.25 times the upper limit of normal range.

Patients with locally advanced breast cancer (other T4 of the 1988 UICC classification), secondary inflammatory breast cancer or metastatic breast cancer (including supraclavicular lymph node involvement) were excluded from the study. The baseline evaluation included physical examination, bilateral mammography and breast echography, chest X-ray, radionuclide bone scan, liver echography, bone marrow aspiration (for further comparison with leukapheresis product) and, if possible, two bone marrow biopsies, standard biological tests and CA 15-3 assay.

Other exclusion criteria were the presence of another concomitant serious illness and an uncontrolled ongoing infection at entry into the study. In accordance with French law, the study was approved by the ethical committee (CCPPRB) of the University of Toulouse and patients had to provide written informed consent before entering the study.

### Treatment plan

Treatment was a combined modality approach including high-dose sequential chemotherapy with rG-CSF (filgrastim) and peripheral blood stem cell support as induction chemotherapy.

#### *Initial local treatment*

Initial surgery was performed to acquire pathological documentation of invasive breast cancer and varied according to each centre policy, from needle biopsy to tumour biopsy with skin biopsy and/or axillary dissection.

#### *Chemotherapy*

Four cycles of chemotherapy were administered every 21 days. Cycle one consisted of cyclophosphamide 6 g $m^{-2}$ and doxorubicin

 © 1999 Cancer Research Campaign

75 mg m$^{-2}$, cycle 2 of cyclophosphamide 3 g m$^{-2}$ and doxorubicin 75 mg m$^{-2}$, cycle 3 and 4 of cyclophosphamide 3 g m$^{-2}$ doxorubin 75 mg m$^{-2}$ and 5-fluorouracil (5-FU) 2500 mg m$^{-2}$. Cyclophosphamide was administered as a 1-h intravenous (i.v.) infusion, dose was divided by two and administered for 2 consecutive days in cycle 1, doxorubicin was given as a 15-min i.v. infusion and 5-FU as a 5-day continuous i.v. infusion. Uromitexan was given in the same dosage as cyclophosphamide, as a 24-h continuous i.v. infusion, starting 1 h before cyclophosphamide. Anti-emetic prophylaxis was assured by anti-$HT_3$ serotonin receptors and corticoid. There was no guideline for salvage treatment.

Semi-saline hyperhydradation (3 l m$^{-2}$ 24 h G 5% with sodium chloride 4.5 g l$^{-1}$ and potassium chloride 1.5 g l$^{-1}$) was started 4 h before cyclophosphamide and stopped 20 h after the end of cyclophosphamide infusion. Uroprotection was assured by uromitexan only at cycle 1. Chemotherapy was administered if absolute neutrophil count (ANC) was $\geq 1.5 \times 10^9$ l$^{-1}$ and platelet count $\geq 100 \times 10^9$ l$^{-1}$. No dose reduction was planned. If patient's neutrophil and platelet count did not meet these criteria on day 21, chemotherapy was delayed until adequate count recovery. Exclusion of patients from the study because of absence of haematological recovery was left to the decision of each investigator.

For doxorubicin and cyclophosphamide, dose intensity was calculated as total chemotherapy administered, divided by body surface area and delay (in weeks) between day 1 of cycle 1 and 3 weeks after day 1 of cycle 4 (or 3 weeks after the theoretical day 1 of cycle 4 for patients who stopped treatment). Relative dose intensity (RDI) was the result of dose intensity divided by theoretical dose intensity.

### *rG-GSF, stem cell collection and reinfusion*

rG-CSF (filgrastim) was administered at a daily dosage of 5 µg kg$^{-1}$ (maximum 300 µg kg$^{-1}$ per day) at each cycle of treatment. Administration started at day 4 of cycle 1 and 2 and day 7 (day of stem cell reinfusion) of cycle 3 and 4. rG-CSF was stopped the day before last apheresis or when ANC reached $0.5 \times 10^9$ l$^{-1}$ on 3 consecutive days for cycles without apheresis.

Apheresis were performed after the first cycle of chemotherapy and/or after the second, depending on the possibilities of each centre. Generally, the procedure was started when the absolute number of CD34+ cells in the peripheral blood rose to 20 µl$^{-1}$. Apheresis were stopped when collected CD34+ cells exceeded $4 \times 10^6$ kg$^{-1}$. Cells were divided into two bags at least, to allow reinfusion of a minimum of $2 \times 10^6$ l$^{-1}$ CD34+ cells kg$^{-1}$ after cycle 3 and cycle 4, after storage in liquid nitrogen.

No attempt was made to purge haematopoietic stem cells of possible tumoural contamination. Haematopoietic stem cells were reinfused on day 7 of cycle 3 and 4, at least 20 h after the end of chemotherapy.

### *Further anticancer therapy*

Mastectomy was performed after induction chemotherapy for non-progressive patients. Locoregional treatment was completed by radiotherapy, according to procedures in each centre. Finally, patients who were menopausal at diagnosis and with positive oestrogen and/or progesterone receptors received Tamoxifene 20 mg day$^{-1}$ for 3 years.

### *Supportive care*

Patients were discharged from hospital after chemotherapy. According to policy in each institution, blood stem cell collection and reinfusion were performed in a conventional hospital unit or in an out patient clinic. When patients became febrile (> 38°C), they were hospitalized to receive i.v. antibiotics. Red blood cells were transfused when haemoglobin was ≤ 8 g dl$^{-1}$ or for anaemia symptoms and platelets were transfused when platelet count was < $20 \times 10^9$ l$^{-1}$ or for haemorrhagic symptoms. All blood products, except blood stem cells, were irradiated at 25 Gy.

**Table 1** Tumour characteristics

| | |
|---|---|
| Extent of inflammatory signs | |
| Limited | 61% |
| Diffuse | 39% |
| N | |
| N0 | 20.2% |
| N1 | 58.5% |
| N2 | 21.3% |
| Pathological classification | |
| Ductal | 80.2% |
| Lobular | 5.5% |
| Other | 14.3% |
| SBR grade | |
| I | 2% |
| II | 30.8% |
| III | 58.2% |
| Non-evaluable | 9% |
| Oestrogen/progesterone receptors | |
| +/+ | 17% |
| +/– or –/+ | 18% |
| –/– | 42% |
| Unknown | 23% |

### Toxicity evaluation

Once per course (D1) physical examination was carried out and Karnofsky index, vital signs, electrocardiogram, complete blood count (CBC) and differential, liver function and creatinin were assessed. During the treatment period, CBC and differential were obtained 3 times a week. At the end of chemotherapy, pretreatment evaluation was repeated, except radionuclide bone scan, marrow aspiration and bone marrow biopsies. Echocardiography or radionuclide cardiac scan was performed at the end of chemotherapy and after radiotherapy. Toxicities were assessed according to WHO criteria.

### Response evaluation

### *Clinical*

Clinical evaluation was performed on day 1 of each cycle of chemotherapy and prior to local treatment. Complete clinical response was defined as clinically complete disappearance of breast inflammation as well as the underlying breast tumour mass. Partial response was at least a 50% decrease in tumour diameter with disappearance of inflammation.

### *Pathological evaluation*

Two independent pathologists performed pathological evaluation using a blind study technique. Pretreatment samples consisted of cytology, incisional biopsy or tumourectomy specimens, and, for some patients, node specimens. Microscopic inspection of pretreatment specimens allowed tumour typing according to WHO classification. Several histological parameters were evaluated.

*© 1999 Cancer Research Campaign* 

**Table 2** Neutropenia and febrile neutropenia

| | Cycle 1 | Cycle 2 | Cycle 3 | Cycle 4 |
|---|---|---|---|---|
| ANC <0.1 × $10^9$ $l^{-1}$ | | | | |
| Frequency | 64% | 41%[a] | 48% | 56% |
| Median duration in days (range) | 4 (1–16) | 3 (1–8) | 5 (1–10) | 4 (1–10) |
| ANC <0.5 × $10^9$ $l^{-1}$ | | | | |
| Frequency | 79% | 75% | 78% | 79% |
| Median duration in days (range) | 5 (1–16) | 4 (1–10) | 5 (1–10) | 5 (1–10) |
| Incidence of febrile neutropenia | 48% | 26%[b] | 48% | 51% |

ANC, absolute neutrophil count. [a]$P$ < 0.05; [b]$P$ < 0.01.

**Table 3** Thrombopenia and transfusions

| | Cycle 1 | Cycle 2 | Cycle 3 | Cycle 4 |
|---|---|---|---|---|
| Plts < 20 × $10^9$ $l^{-1}$ | | | | |
| Frequency | 41% | 26%[a] | 44% | 46% |
| Median duration in days (range) | 2 (1–15) | 1 (1–27) | 2 (1–11) | 2 (1–12) |
| Plts < 50 × $10^9$ $l^{-1}$ | | | | |
| Frequency | 63% | 56% | 70% | 69% |
| Median duration in days (range) | 4 (1–15) | 3 (1–33) | 5 (1–27) | 5 (1–14) |
| Incidence of plt transfusions | 43% | 29%[b] | 53% | 56% |
| Median no. of transfusions (range) | 1 (1–6) | 1 (1–6) | 2 (1–5) | 1 (1–4) |
| Incidence of RBC transfusions | 37% | 38% | 66% | 83% |
| Median no. of transfusions (range) | 1 (1–6) | 1 (1–6) | 2 (1–8) | 1 (1–4) |

Plts, platelets. [a]$P$ < 0.05; [b]$P$ < 0.01.

Hormonal receptors were evaluated using immunohistochemistry or biochemical assay.

Mastectomy specimens were thoroughly examined, with sections taken from each quadrant, from the nipple areolar complex and areas suspected of having tumour involvement. A minimum of 20 samples was done. Three components were systematically evaluated: intraductal, invasive carcinoma and vascular invasion. Response in the breast was defined as described by Chevallier et al (1995):

- Grade 1: disappearance of all tumour both on macroscopic and microscopic examination
- Grade 2: presence of in situ carcinoma of the breast with no invasive tumour
- Grade 3: presence of invasive carcinoma with stromal alterations such as sclerosis or fibrosis
- Grade 4: no or few alterations in tumoural appearance.

Lymph nodes were evaluated separately when available after chemotherapy and classified in two categories: involved or not involved.

### Statistical analysis

No interim analysis on efficacy was performed. However, it was planned to stop the study if toxic death rate was too high. To keep toxic death rate under 3% with a 5% $\alpha$ risk, only up to four toxic deaths among the first 30 patients were accepted (Fleming, 1982).

Medians are presented with their range and response rate with 95% confidence interval. Percentage differentials were tested by application of the $\chi^2$ test. When a patient stopped her treatment, she was analysed for received cycle toxicity, dose intensity and pathological response if mastectomy was performed before beginning another antineoplasic treatment and for follow-up.

**Table 4** Response

| | Number of evaluable patients | Percentage of responders |
|---|---|---|
| Objective clinical response | 94 | 90% ± 6% |
| Pathological response in breast | 87 | GR I and II: 32% ± 10% |
| | | GR III and IV: 68% ± 10% |

GR I = Disappearance of tumour both on macroscopic and microscopic examination. GR II = Presence of in situ carcinoma of the breast, with no invasive tumour. GR III = Presence of invasive carcinoma with stromal alterations such as sclerosis or fibrosis. GR IV = No or few alterations of tumoural appearance.

Survival and relapse-free survival were estimated using the Kaplan–Meier method (Kaplan et al, 1971). Relapse-free survival was defined as the time elapsed between date of diagnosis and date of first relapse, wherever this relapse might be. Overall survival was the period between time of diagnosis and time of last status report, whether the patient was alive or dead, whatever the cause of death.

### RESULTS

### Patients

Between December 1994 and September 1996, 100 patients from 17 participating centres entered the study. Five patients were withdrawn from the study: four had metastatic inflammatory breast cancer at diagnosis (positive radionuclide bone scan: two, contralateral or supraclavicular lymph nodes: two) and were not eligible and one received another chemotherapy regimen before the first cycle. Finally, 95 patients were valid for analysis.

Median age of patients was 46 years (range 26–59), 83.2% were premenopausal at time of diagnosis. Initial characteristics of tumours are summarized in Table 1. Axillary dissection was initially performed in only 17 patients. Median number of involved nodes for these patients was 8 (range 0–23), with eight patients having ten or more involved nodes. Dermal lymphatic carcinomatosis was found in 43% of patients who had a skin biopsy.

### Stem cell collection and infusion

Ninety-seven per cent of patients had successful collection of CD34+ cells after cycle 1 and/or cycle 2, and 93% of all patients after 1 single set of apheresis. Median number of collected CD34+ cells was 14.75 × $10^6$ $kg^{-1}$ (range 2.3 to > 100), and a median of 6.05 × $10^6$ $kg^{-1}$ (range 1.2 to >100) and 8.5 × $10^6$ $kg^{-1}$ (range 1.2–59.1) CD34+ cells were respectively reinfused after cycle 3 and cycle 4.

### Toxicity

### *Non-haematologic toxicity*

Grade 3 or 4 vomiting occurred in 14% of cycles, grade 3 or 4 mucositis in 10% of cycles (4% in cycles 1 and 2, 15% in cycle 3 and 4, $P$ < 0.01). Grade 3 hepatic toxicity was seen in one single patient in cycle 1 and in another patient in cycle 3. No other grade 3

© 1999 Cancer Research Campaign

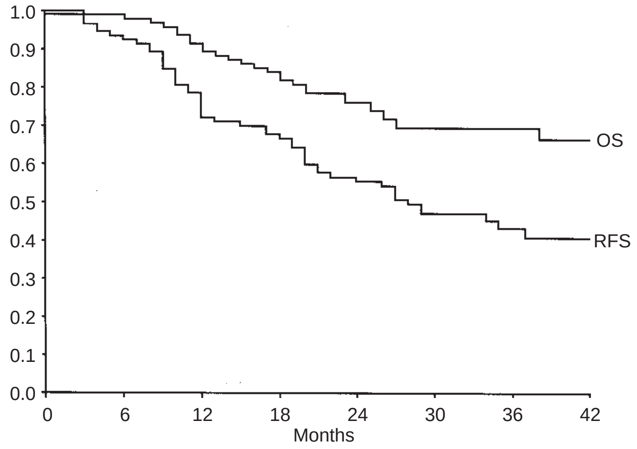

**Figure 2** Survival (OS) and relapse-free survival (RFS).

or 4 toxicities were seen during the study. Monitoring of left ventricular ejection fraction showed no clinically significant diminution.

### Haematologic toxicities

*Neutropenia and rehospitalization*

One patient died from the procedure. She was readmitted for febrile neutropenia after cycle 1 and died from septic shock with multi-organ failure. Grade 4 neutropenia occurred in between 75% and 79% of each cycle. Duration of neutropenia inferior to $0.5 \times 10^9$ $l^{-1}$ lasted a median of 5 days per cycle. Febrile neutropenia was the most frequent reason for rehospitalization (85% of all rehospitalizations). Overall, there was no cumulative increase in frequency and duration of neutropenia and complications over the four cycles. However, cycle 2 was overall significantly associated with less toxicity (Table 2). Emergency readmission was necessary in 51% of the 366 administered cycles. The median duration of rehospitalization was: 6.5 days (range 1–16), 5 days (range 1–29), 8 days (range 1–19) 6 days (range 2–16) for each cycle.

*Thrombopenia and transfusion (Table 3)*

Thrombopenia of less than $20 \times 10^9$ $l^{-1}$ occurred in 26–46% of cycles. Duration of thrombopenia inferior to $20 \times 10^9$ $l^{-1}$ was a median 2 days per cycle, with the same duration after each cycle. Platelets transfusion was needed in an average of 29–56% of cycles with administration of a median of one transfusion per cycle.

### Chemotherapy delivery

A total of 366 cycles of chemotherapy were administered in 95 evaluable patients. Ninety-three patients received the 2nd cycle, 91 the 3rd cycle and 87 the 4th. Except for the patient who died after cycle 1, the main reason for stopping chemotherapy was prolonged haematological toxicity.

Cycle 2 was administered in a median of 21 days (range 18–37) after cycle 1, cycle 3 in a median of 21 days (range 19–35) after cycle 2 and cycle 4 in a median of 21 days (range 20–43) after cycle 3. Cycles 2, 3 and 4 were respectively delayed more than 1 week for four, nine and 11 patients.

Median received dose intensity for cyclophosphamide and doxorubicin was 1211 mg $m^{-2}$ $week^{-1}$ (range 50–1305) and 24 mg $m^{-2}$ $week^{-1}$ (range 6–26) with respective relative dose intensity of 0.97 (range 0.40–1.04) and 0.96 (range 0.25–1.05).

### Anti-tumoural response (Table 4)

*Clinical response*

Out of 94 evaluable patients, one did not have a clinical response after four cycles of chemotherapy (persistence of inflammatory signs). All 93 other patients had good clinical response to chemotherapy with complete disappearance of tumoural signs (clinical complete response) in 75 (80%). After the 1st cycle of chemotherapy, inflammatory signs disappeared in 34 patients (41%).

*Pathological response*

One patient, with persistence of inflammatory signs, did not undergo mastectomy and was evaluated as a pathological failure. For eight other patients with clinical complete response, mastectomy was not performed at the patients' request. Conservative treatment was given to these patients. These eight patients were not evaluated for pathological response. Finally, 86 patients underwent mastectomy, which was performed in a median of 3.5 months (range 3–9) after the first cycle of chemotherapy: 28 patients experienced complete disappearance of tumour cells (grade I) or only persistence of an intraductal component (grade II) (32 ± 10% of grade I or II pathological response). In 24 other patients (28 ± 9%), major changes in histology were found, such as tumour cell necrosis and stromal alteration showing partial efficacy of chemotherapy (grade III). However, an invasive component was persistent in these patients. Finally, in 34 patients (39 ± 10%), despite good clinical response, no evidence of pathological response to chemotherapy (grade IV) was seen.

Objective evaluation of response rate in breast and lymph nodes was difficult to assess since several patients had pathological complete response in the breast, but had undergone previous axillary dissection. Among the 69 patients who underwent axillary dissection after chemotherapy, 18 (26 ± 10%) had negative lymph node.

As clinical complete response was present in 80% of patients, it was not possible to make any correlation between clinical and pathological complete response.

*Survival*

With a 3-year median follow-up, 29 patients died and 66 are alive. The estimated 3-year survival is 70% (95% confidence interval (CI) 60–79%) and the median survival is not reached. Fifty-two relapses occurred (43 distant and nine local) with a 3-year relapse-free survival of 44% (95% CI 33–54%).

### DISCUSSION

This study was initially designed to evaluate feasibility of high-dose sequential chemotherapy with rG-CSF and blood stem cell transplantation in inflammatory breast cancer, to test its impact on response rate and possibly outcome. As response, and particularly pathological response (Feldman et al, 1986; Noguchi et al, 1988; Maloisel et al, 1990; Armstrong et al, 1993; Palangie et al, 1994; Sataloff et al, 1995), have been described as main prognostic

© 1999 Cancer Research Campaign 

**Table 5** Pathological response rate: comparison with conventional chemotherapy

| Author | Patient no. | Chemotherapy | Microscopic response rate (%) |
|---|---|---|---|
| Feldman (*Cancer Res*, 1986) | 90 | 5FU–D–Cy | CR: 7 |
| Israël (*Cancer*, 1986) | 24 | 5FU–Cy | CR: 0<br>‘Major response’: 17 |
| Noguchi (*Cancer*, 1988) | 28 | MMC–5FU or D (Intra arterial) | CR: 25 |
| Maloisel (*Cancer*, 1990) | 44 | D–5FU–Cy | CR: 18 |
| Armstrong (*Breast Cancer Res Treat*, 1993) | 24 | D–Cy- VC–MTX–L-5FU | CR: 17 |
| Chevallier (*J Clin Oncol*, 1995) | 97 | 5FU–Ep–Cy ± Lenograstim | CR: 22 |
| Colozza (*Am J Clin Oncol*, 1996) | 31 | CDDP–D–Cy | CR: 8 |
| Present study | 87 | Cy-D-5FU High dose | CR: 32 |

D: doxorubicin, Cy: cyclophosphamide, MMC: mitomycin-c, VC: vincristine, 5-FU: 5-fluorouracil, MTX: methotrexate, L: leucovorin, Ep: epirubicin, CDDP: cisplatin, CR: complete response.

factors in inflammatory breast cancer, pathological response rate was considered as an acceptable early end point to test efficacy of this new chemotherapy regimen.

Acute toxicity related to chemotherapy consisted of mainly severe but reversible pancytopenia, occurring in all four cycles of chemotherapy. The second cycle of chemotherapy was less toxic, leading to fewer cases of severe neutropenia, febrile neutropenia, thrombopenia and platelet transfusion. This difference was expected, since cycle 2 differed from cycle 1 in cyclophosphamide dose (3 g $m^{-2}$ vs 6 g $m^{-2}$) and from cycles 3 and 4 by absence of 5-FU.

The relatively short duration of neutropenia is probably related to use of rG-CSF. One can question the utility of peripheral blood stem cells in this study, in which there was no myeloablative chemotherapy. However, it can be noted that the incidence of severe neutropenia, thrombopenia and febrile neutropenia did not increase from cycle 1 to cycle 4. In previously published studies of high-dose doxorubicin-cyclophosphamide regimen with rG-CSF but without stem cell transplantation (Shipp et al, 1995; Swain et al, 1996), thrombocytopenia is generally the dose-limiting toxicity, this appears to be cumulative and increases significantly between the first and last cycle (Shipp et al, 1995). These toxicities occur in the same range of doses as those used in our study: cyclophosphamide 2000 mg $m^{-2}$; doxorubicin 40 mg $m^{-2}$ every 2 weeks (Swain et al, 1996); cyclophosphamide 4000 mg $m^{-2}$; doxorubicin 70 mg $m^{-2}$ every 3 weeks for 4 cycles (Shipp et al, 1995).

In other studies using additional blood cells with high-dose chemotherapy (Basser et al, 1995; Stoppa et al, 1997; Viens et al, 1997) even if it is cumulative (Basser et al, 1995; Stoppa et al, 1997) thrombocytopenia seems to be less severe and the planned dose intensity is easily respected (Basser et al, 1995; Viens et al, 1997). Overall, using peripheral blood stem cells seems to permit a safer and more regular increase of dose intensity in high-dose sequential chemotherapy regimens.

One of the risks in using peripheral blood stem cells is the mobilization, collection and reinfusion of tumour cells (Brugger et al, 1994). This is a potential risk in our study where most patients had blood stem cell collection after the first cycle of chemotherapy. However, the significance of circulating tumour cells and impact of their potential reinfusion are not yet clearly established, and ex vivo therapy is not considered presently as a standard practice. So, it was decided in the context of such a multicentric study of first-line chemotherapy, to avoid purging and plan secondary analysis of the possible impact of tumoural contamination on progression-free survival rather than on response, which was the main objective of this study.

Among the 100 patients, one fatality was observed due to septic shock during neutropenia. This death is clearly related to the therapy, but overall, treatment-related mortality rate (≈1%) remains in the lower range of those reported in trials of high-dose chemotherapy with stem cell transplantation.

Non-haematologic toxicities were essentially mucositis, occurring more frequently after cycle 3 and 4 probably related to administration of 5-FU.

Relative dose intensity was 0.97 (range 0.4–1.04) for cyclophosphamide and 0.96 (range 0.25–1.05) for doxorubicin. Eighty-seven patients received four cycles of chemotherapy, i.e. 15 g $m^{-2}$ of cyclophosphamide, 300 mg $m^{-2}$ of doxorubicin and 5000 mg $m^{-2}$ of 5-FU in 9 weeks. This strategy of high-dose sequential chemotherapy with stem cell support allows a total dose of cyclophosphamide that is around 7.5 times higher than that received in treatment which associates four cycles of standard FAC (Feldman et al, 1986) and 3 times higher than in the FEC high dose described by Chevallier et al (1995). Our data show that such an increase in dose and dose intensity is accessible for 92% of patients with non-metastatic inflammatory breast cancer with the use of rG-CSF and peripheral stem cells.

The second end point of our study was to evaluate response rate of inflammatory breast cancer to a dose-intensified cyclophosphamide-doxorubicin 5-FU regimen. Clinical response rate was high (OR: 90%), as generally described with other anthracyclin-based regimens. When pathological response in breast was considered in 87 evaluable patients, only 32% had total disappearance of invasive tumoural cells. Several pathological response rates, comparably defined, have been previously published after conventional or moderately intensified systemic induction chemotherapy (Table 5). Feldman et al (1986), using standard FAC reported 12% of pathological complete response in breast among 90 patients. Chevallier et al (1995) using an intensified FEC reported 22% of pathological complete response in 97 patients. In other studies, where smaller numbers of patients were reported

 *© 1999 Cancer Research Campaign*

(inferior to 50), complete pathological response rate ranged from 0% to 18% after various combination chemotherapies (Israel et al, 1986; Maloisel et al, 1990; Armstrong et al, 1993; Colozza et al, 1996). Our results show a relatively higher pathological response rate when compared to large series in the literature; however, it remains limited, compared to the major increase in dose intensity and total dose of chemotherapy, particularly of cyclophosphamide (15 g $m^{-2}$) which consequently resulted in an important increase in toxicities and hospitalization. Is this because the main dose increase was with cyclophosphamide? Recently, the NSABP (Fisher et al, 1997) showed that a dose escalation of cyclophosphamide from 2400 mg $m^{-2}$ to 4800 mg $m^{-2}$ in 9 weeks did not result in any benefit in the adjuvant situation. However, dose escalation was much higher in our study, also IBC and adjuvant situation are different, consequently extrapolation of the NSABP results to our study is difficult.

Chemotherapy regimen used in our study resulted essentially in an increase of dose intensity with a relatively moderate increase in dose, being the opposite of the general procedure in high-dose chemotherapy with stem cell transplantation, which raises the question of dose–effect versus dose–intensity. High pathological response rate is generally reported with high-dose chemotherapy with stem cell support in inflammatory breast cancer, but data are available only for a very small number of studies and patients: two pathological complete responses in two patients (Nieto et al, 1997), four pathological complete responses among nine patients (Rosti et al, 1997), seven pathological complete responses in 18 mastectomies (Viens et al, 1998). Furthermore, most of these patients were selected for response to standard chemotherapy prior to intensification, which excludes analysis for poor responder patients. Contrary to these studies, first-line high-dose sequential chemotherapy, like ours, could be valuable in a large proportion of patient, with untreated IBC. Similar encouraging results have been reported with such a strategy in metastatic disease (Bezwoda et al, 1995) and in the adjuvant situation (Gianni et al, 1997) but using generally higher dosages of alkylating agents. According to the increase of pathological response rate, the 3-year survival is encouraging, but the benefit cannot be definitely established on that phase II study. More studies are needed to optimize the chemotherapy sequence including other drugs, other escalation and/or combinations.

Finally, the benefit of this approach needs to be prospectively evaluated, in a randomized fashion against standard chemotherapy, considering survival, cost and quality of life.

### ACKNOWLEDGEMENTS

The authors thank the Department of Biostatistics of the Institut Curie and B Asselain and M Barrand for their help in collecting and analysing data. This work as the whole PEGASE programme received special grants from the French Administration of Health and the Ligue Nationale contre le Cancer. As was the whole PEGASE programme, this trial is supported by the French Federation of Anti-cancer Center (FNCLCC). Additional grants were given by Amgen France/Produits Roche, Pharmacia-Upjohn and Wyeth-Lederle.

### REFERENCES

Antman K and Gale RP (1988) Advanced breast cancer: high-dose chemotherapy and bone marrow autotransplants. *Ann Intern Med* **108**: 570–574

Armstrong DK, Fetting JH, Davidson NE, Gordon GB, Huelskamp AM and Abeloff MD (1993) Sixteen-week dose intense chemotherapy for inoperable, locally advanced breast cancer. *Breast Cancer Res Treat* **28**: 277–284

Basser RL, To LB, Begley CG, Juttner CA, Maher DW, Szer J, Cebon J, Russel I, Olver I, Gill PG, Fox RM, Sheridan WP and Green MD (1995) Adjuvant treatment of high-risk breast cancer using multicycle high-dose chemotherapy and filgrastim-mobilized peripheral blood progenitor cells. *Clin Cancer Res* **1**: 715–721

Bauer RL, Busch E, Levine E and Edge SB (1995) Therapy for inflammatory breast cancer: impact of doxorubicin-based therapy. *Ann Surg Oncol* **2**: 288–294

Bezwoda WR, Seymour L and Dansey RD (1995) High-dose chemotherapy with hematopoietic rescue as primary treatment for metastatic breast cancer: a randomized trial. *J Clin Oncol* **13**: 2483–2489

Brugger W, Bross KJ, Glatt M, Weber F, Mertelsmann R and Kanz L (1994) Mobilization of tumor cells and hematopoietic progenitor cells into peripheral blood of patients with solid tumors. *Blood* **83**: 636–640

Chevallier B, Asselain B, Kunlin A, Veyret C, Bastit P and Graic Y (1987) Inflammatory breast cancer. Determination of prognostic factors by univariate and multivariate analysis. *Cancer* **60**: 897–902

Chevallier B, Chollet P, Merrouche Y, Roche H, Fumoleau P, Kerbrat P, Genot JY, Fargeot JP, Olivier JP, Fizames C, Clavel M, Yver M and Cour Chabernaud, V (1995) Lenograstim prevents morbidity from intensive induction chemotherapy in the treatment of inflammatory breast cancer. *J Clin Oncol* **13**: 1564–1571

Colozza M, Gori S, Mosconi AM, Anastasi P, De Angelis V, Giansanti M, Mercati U, Aristei C, Latini P and Tonato M (1996) Induction chemotherapy with cisplatin, doxorubicin, and cyclophosphamide (CAP) in a combined modality approach for locally advanced and inflammatory breast cancer. Long-term results. *Am J Clin Oncol* **19**: 10–17

Feldman LD, Hortobagyi GN, Buzdar AU, Ames FC and Blumenschein GR (1986) Pathological assessment of response to induction chemotherapy in breast cancer. *Cancer Res* **46**: 2578–2581

Fisher B, Anderson S, Wickerham DL, DeCillis A, Dimitrov N, Mamounas E, Wolmark N, Pugh R, Atkins JN, Meyers FJ, Abramson N, Wolter J, Bornstein RS, Levy L, Romond EH, Caggiano V, Grimaldi M, Jochimsem P and Deckers P (1997) Increased intensification and total dose of cyclophosphamide in a doxorubicin-cyclophosphamide regimen for the treatment of primary breast cancer: findings from national surgical adjuvant breast and bowel project B-22. *J Clin Oncol* **15**: 1858–1869

Fleming TR (1982) One-sample multiple testing procedure for phase II clinical trials. *Biometrics* **38**: 143–151

Frei E III and Canellos GP (1980) Dose: a critical factor in cancer chemotherapy. *Am J Med* **69**: 585–594

Gianni AM, Siena S, Bregni M, Di Nicola M, Orefice S, Cusumano F, Salvadori B, Luini A, Greco M, Zucali R, Rilke F, Zambetti M, Valagussa P and Bonadonna G (1997) Efficacy, toxicity, and applicability of high-dose sequential chemotherapy as adjuvant treatment in operable breast cancer with 10 or more involved axillary nodes: five-year results. *J Clin Oncol* **15**: 2312–2321

Griswold DP Jr, Trader MW, Frei EI, Peters WP, Wolpert MK and Laster WR Jr (1987) Response of drug-sensitive and resistant L1210 leukemias to high-dose chemotherapy. *Cancer Res* **47**: 2323–2327

Hryniuk W and Bush H (1984) The importance of dose intensity in chemotherapy of metastatic breast cancer. *J Clin Oncol* **2**: 1281–1288

Israel L, Breau J and Morere J (1986) Two years of high-dose cyclophosphamide and 5-fluorouracil followed by surgery after 3 months for acute inflammatory breast carcinomas. A phase II study of 25 cases with a median follow-up of 35 months. *Cancer* **57**: 24–28

Jaiyesimi IA, Buzdar AU and Hortobagyi GN (1992) Inflammatory breast cancer: a review. *J Clin Oncol* **10**: 1014–1024

Kaplan EL and Meier P (1971) Non parametric estimation from incomplete observations. *J Am Stat Assoc* **53**: 457–481

Maloisel F, Dufour P, Bergerat JP, Herbrecht R, Duclos B, Boilletot A, Giron C, Jaeck D, Haennel P, Jung G and Oberling F (1990) Results of initial doxorubicin, 5-fluorouracil, and cyclophosphamide combination chemotherapy for inflammatory carcinoma of the breast. *Cancer* **65**: 851–855

Nieto Y, Cagnoni PJ, Bearman SI, Shpall EJ, Ross M and Jones RB (1997) High-dose chemotherapy (HDC) with cisplatin (CDDP), cyclophosphamide (CPA) and BCNU (CCB), followed by autologous hematopoietic progenitor cell support (AHPCS) for inflammatory breast cancer (IBC). In *Proceedings of Asco* Vol. 16. pp. 436: Denver, CO.

Noguchi S, Miyauchi K, Nishizawa Y, Koyama H and Terasawa T (1988) Management of inflammatory carcinoma of the breast with combined modality therapy including intraarterial infusion chemotherapy as an induction therapy. *Cancer* **61**: 1483–1491

Palangie T, Mosseri V, Mihura J, Campana F, Beuzeboc P, Dorval T, Garcia-Giralt E, Jouve M, Scholl S, Asselain B and Pouillart P (1994) Prognostic factors in inflammatory breast cancer and therapeutic implications. *Eur J Cancer* **30A**: 921–927

Peters WP, Ross M, Vredenburgh JJ, Meisenberg B, Marks LB, Winer E, Kurtzberg J, Bast RC Jr, Jones R, Shpall E, Wu K, Rosner G, Gilbert C, Mathias B, Coniglio D, Petros W, Henderson IC, Norton L, Weiss RB, Budman DR and Hurd D (1993) High-dose chemotherapy and autologous bone marrow support as consolidation after standard-dose adjuvant therapy for high-risk primary breast cancer. *J Clin Oncol* **11**: 1132–1143

Rosti G, Vertogen B, Ferrante P, Turazza M, Lelli G, Sabbatini R, Frassineti GL, Tienghi A and Marangolo M (1997) High-dose mitoxantrone, thiotepa and cyclophosphamide with peripheral blood progenitor cells (PBPC) support for inflammatory breast carcinoma; an Italian study. In *Proceedings of Asco*, Vol. 16. pp. 125: Denver, CO

Rouëssé J, Friedman S, Sarrazin D, Mouriesse H, Le Chevalier T, Arriagada R, Spielmann M, Papacharalambous A and May-Levin F (1986) Primary chemotherapy in the treatment of inflammatory breast carcinoma: a study of 230 cases from the Institut Gustave Roussy. *J Clin Oncol* **4**: 1765–1771

Sataloff DM, Mason BA, Prestipino AJ, Seinige UL, Lieber CP and Baloch Z (1995) Pathologic response to induction chemotherapy in locally advanced carcinoma of the breast: a determinant of outcome. *J Am Coll Surg* **180**: 297–304

Shipp MA, Neuberg D, Janicek M, Canellos GP and Shulman LN (1995) High-dose CHOP as initial therapy for patients with poor-prognosis aggressive non-Hodgkin's lymphoma: a dose-finding pilot study. *J Clin Oncol* **13**: 2916–2923

Stoppa AM, Bouabdallah R, Chabannon C, Novakovitch G, Vey N, Camerlo J, Blaise D, Xerri L, Resbeut M, Di Stefano D, Bardou VJ, Gastaut JA and Maraninchi D (1997) Intensive sequential chemotherapy with repeated blood stem-cell support for untreated poor-prognosis non-Hodgkin's lymphoma. *J Clin Oncol* **15**: 1722–1729

Swain SM and Lippman ME (1989) Treatment of patients with inflammatory breast cancer. In: *Important Advances in Oncology*, De Vita VT Jr, Rosenberg SA (ed), pp. 129–150. Lippincott: Philadelphia, PA

Swain SM, Rowland J, Weinfurt K, Berg C, Lippman ME, Walton L, Egan E, King D, Spertus I and Honig SF (1996) Intensive outpatient adjuvant therapy for breast cancer: results of dose escalation and quality of life. *J Clin Oncol* **14**: 1565–1572

Viens P, Gravis G, Genre D, Bertucci F, Cowen D, Camerlo J, Cappiello MA, Conte M, Finaud M, Chabannon C, Houvenaeghel G and Maraninchi D (1997) High-dose sequential chemotherapy with stem cell support for non-metastatic breast cancer. *Bone Marrow Transplant* **20**: 199–203

Viens P, Penault-Llorca F, Jacquemier J, Gravis G, Cowen D, Bertucci F, Houvenaeghel G, Blaise D and Maraninchi D (1998) High-dose chemotherapy and haematopoietic stem cell transplantation for inflammatory breast cancer: pathologic response and outcome. *Bone Marrow Transplant* **21**: 249–254

*© 1999 Cancer Research Campaign*
